# Coordinate Regulation of Cholesterol and Bile Acid Metabolism by the Clock Modifier Nobiletin in Metabolically Challenged Old Mice

**DOI:** 10.3390/ijms20174281

**Published:** 2019-09-01

**Authors:** Kazunari Nohara, Travis Nemkov, Angelo D’Alessandro, Seung-Hee Yoo, Zheng Chen

**Affiliations:** 1 Department of Biochemistry and Molecular Biology, The University of Texas Health Science Center at Houston, 6431 Fannin St., Houston, TX 77030, USA; 2 Department of Biochemistry and Molecular Genetics, University of Colorado Denver—Anschutz Medical Campus, Aurora, CO 80045, USA

**Keywords:** circadian clock modifier, Nobiletin, cholesterol and bile acid homeostasis, gut microbiota, liver, gene expression, aging, high-fat feeding

## Abstract

Cholesterol and bile acid (BA) homeostasis plays a central role in systemic metabolism. Accumulating evidence suggests a key regulatory function of the circadian clock, our biological timer, in lipid metabolism, particularly cholesterol and bile acid flux. Previously, we showed that Nobiletin (NOB), a natural compound targeting the ROR (Retinoic acid receptor-related orphan receptor) nuclear receptors in the circadian oscillator, strongly protects lipid homeostasis, including normal serum cholesterol levels in high-fat (HF) fed mice at both young and old ages. In this study, we further examined the role of NOB in cholesterol metabolism in HF-fed aged mice, and found that NOB lowered the serum LDL/VLDL cholesterol levels and consequently the LDL/HDL ratio. BA levels in the serum were markedly reduced in the HF.NOB group, and examination of additional hepatic markers further indicate a protective role of NOB in the liver. At the molecular level, whereas HF feeding downregulated hepatic expression of several ROR target genes involved in bile acid synthesis, NOB treatment (HF.NOB) was able to rescue it. In accordance, fecal BA excretion was enhanced by NOB, and microbial 16S sequencing revealed alteration of several taxa known to be involved in secondary BA production in the gut. Together, these results demonstrate concerted effects of the clock-modulating compound NOB in cholesterol and BA metabolism, suggesting pharmacological manipulation of the clock as a novel therapeutic strategy against metabolic disorders and age-related decline.

## 1. Introduction

Cholesterol and bile acid homeostasis plays a central role in metabolic health. Cholesterols are essential components in the plasma membrane, and serve as precursors for critical regulators of homeostasis such as steroid hormones and bile acids [1]. Bile acids (BAs) function as emulsifiers for dietary lipids and also in cellular signaling as ligands for nuclear and G-protein coupled receptors to regulate metabolism, immunity and other physiological processes [2]. BA synthesis from cholesterol by hepatic cytochrome P450s (CYPs) is a critical node in energy metabolism. Following synthesis in the liver, BAs are temporarily stored in the gallbladder, and released into the duodenum upon meals where they facilitate micelle formation and emulsion. Thereafter, primary BAs are metabolized by enzymes from gut microbiota to produce secondary BAs, and approximately 90–95% of BAs are reabsorbed in the ileum and transported back to the liver via the portal vein [3,4]. Daily hepatic production and fecal excretion of BAs each account for approximately 5% of total BA levels. Therefore, cholesterol and BA metabolism is highly complex, involving multiple organs and intestinal microbiota, and the levels in the circulation vary depending on meals and physiological factors.

A key regulatory mechanism of cholesterol and BA metabolism is the circadian clock, our intrinsic biological timer. The clock is an ensemble of cell-autonomous oscillators orchestrated by the master pacemaker, the suprachiasmatic nuclei in the hypothalamus [5,6,7]. The oscillator itself consists of intersecting negative feedback loops, each loop containing positive (including CLOCK/NPAS2, BMAL1, RORs) and negative components (CRYs, PERs, REV-ERBs) [5]. The cellular oscillators broadly drive the expression of clock-controlled genes throughout the body, thereby controlling metabolic and physiological processes in virtually all cells and tissues [8,9,10,11]. Beyond the mammalian host tissues, recent studies have also reveal a crosstalk between circadian machinery and microbial dynamics, which oscillate in a time-of-day-dependent manner [12,13,14]. As we age, circadian gene expression and physiological rhythms undergo progressive changes, notably, a dampening of rhythmic amplitude and a worsened response to entraining cues [15,16,17,18]. In accordance, circadian disruption has been shown to aggravate mortality and morbidity in mice [19,20].

Growing evidence highlights the pivotal function of the circadian clock in lipid homeostasis, including cholesterol and BA metabolism [21,22,23,24,25,26,27]. Circadian disruption by various environmental and genetic means has been found to perturb cholesterol and BA homeostasis [28,29,30,31,32]. Previously, genomic and molecular studies revealed the key role of REV-ERBα, the negative factor in the circadian secondary loop, in SREBP-mediated regulation of cholesterol and lipid metabolism [33]. Interestingly, it was shown that REV-ERBα activated the expression of the *Cyp7a1* gene encoding cholesterol-7alpha-hydroxylase, the rate-limiting enzyme in bile acid synthesis, likely via an indirect mechanism. Consistent with a key role of the secondary loop in cholesterol/BA metabolism, multiple studies have revealed that the retinoid acid receptor related orphan receptors (RORs), the positive factor opposing REV-ERB function, controls important genes in cholesterol/BA homeostasis, including *Cyp7b1* and *Cyp8b1* known to be involved in classical and alternative BA synthetic pathways [34,35,36,37,38,39,40]. Notably, various endogenous ligands, mainly cholesterol derivatives and related intermediate metabolites, have been shown to bind to the RORα and RORγ subtypes [41,42,43], underscoring a potential feedback regulation between RORs and lipid homeostasis.

We previously conducted chemical screening and identified a naturally occurring compound, Nobiletin (NOB), that enhances circadian rhythms and protects against metabolic disorders [44,45]. In filter binding and cellular assays, NOB was found to serve as an agonist of RORs [44]. This was consistent with the robust efficacy of NOB to improve lipid homeostasis, including serum cholesterol, and remodel hepatic gene regulatory pathways in high fat (HF)-fed obese mice. More recently, NOB has been found to also enhance serum homeostasis of lipids, including cholesterol in naturally aged mice subjected to the metabolic challenge of HF feeding [46]. Together, these results suggest a possible role of NOB, as a natural ROR agonist, in cholesterol and BA homeostasis. In the current study, we further examined cholesterol and BA profiles in HF-fed aged mice, and uncovered a role of NOB to improve multiple serum cholesterol and BA parameters, protect liver health and BA-related gene regulation, increase fecal BA secretion and remodel gut microbiota. Together, our study underscores a coordinated regulatory role of the clock modulator NOB in cholesterol and BA metabolism.

## 2. Results

### 2.1. Nobiletin Improves Serum Cholesterol Profiles in Aged Mice under High-Fat Feeding

Our previous study showed NOB treatment improved serum and hepatic lipid profiles in metabolic disease mouse models, particularly HF-fed obese mice [44]. More recently, in aged mice, we observed that levels of serum lipids including triglyceride, free glycerol and free fatty acid were significantly attenuated by NOB treatment in HF-fed aged mice (HF.NOB) [46]. To further investigate cholesterol homeostasis in HF-fed aged mice, we performed colorimetric ELISA assays using serum samples collected during the day- and night-time (zeitgeber time ZT6 and ZT18, respectively).

**Figure 1:**
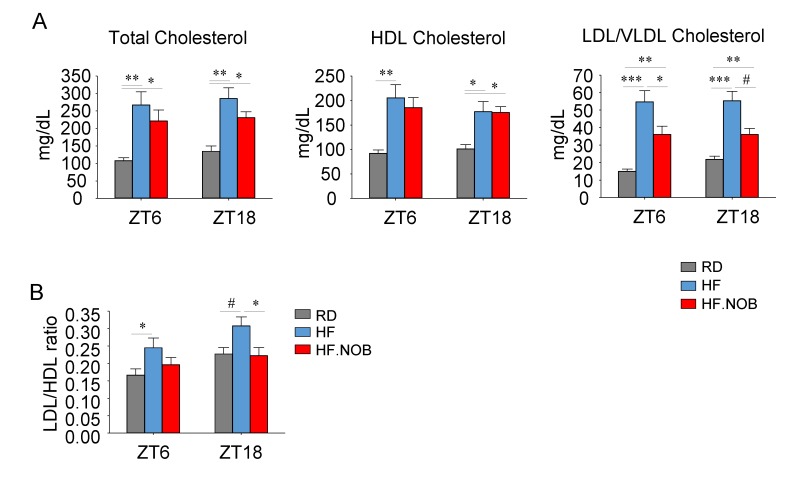
Nobiletin (NOB) improves cholesterol homeostasis in aged mice. (**A**) Serum total cholesterol levels, HDL cholesterol levels, LDL/VLDL cholesterol levels, and (**B**) LDL/HDL ratios were measured by colorimetric assays (*n* = 6–8). RD: regular diet; HF: high-fat diet; HF.NOB: high-fat diet with 0.1% NOB. *p < 0.05, **p < 0.01, ***p < 0.001, One-Way ANOVA; #*p* < 0.05, t-test. Bar graphs represent Mean ± SEM.

Our results showed that the total HDL and LDL/VLDL cholesterol levels were all increased in the HF group relative to regular diet (RD) controls (Figure 1A). Interestingly, NOB markedly suppressed LDL/VLDL cholesterol levels, whereas HDL cholesterol levels were not significantly decreased (Figure 1A). As a result, NOB attenuated the LDL/HDL ratio, a key metabolic parameter known to be exaggerated in metabolic/cardiovascular diseases [47] (Figure 1B). Consistent with our previous observations [44], these results illustrate the beneficial role of NOB for cholesterol homeostasis in the serum.

### 2.2. Nobiletin Attenuates Bile Acids Leakage in the Serum and Safeguards Healthy Liver

**Figure 2:**
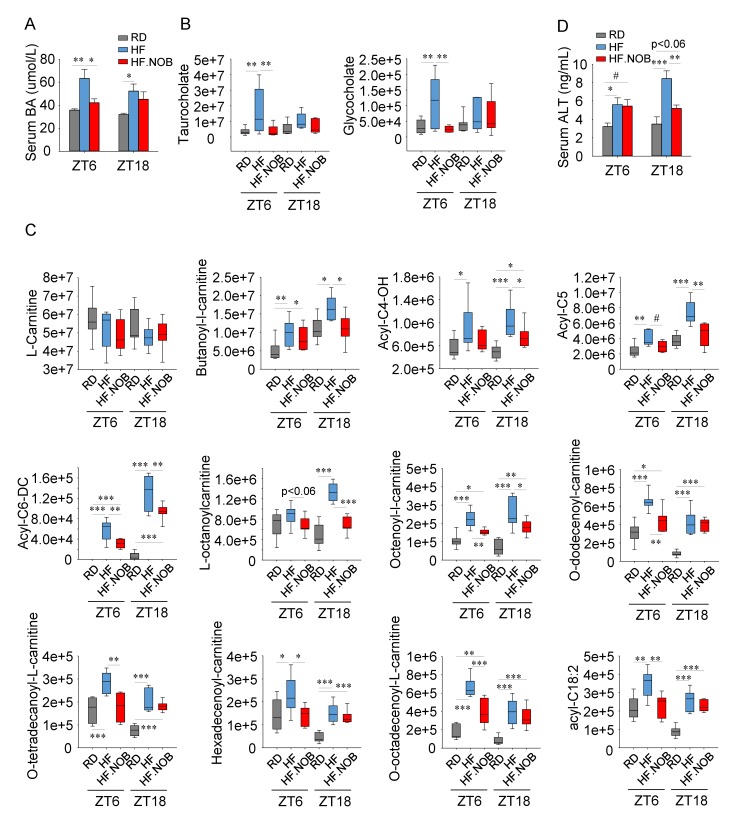
Nobiletin attenuates serum bile acid levels and other hepatic damage markers. (**A**) Serum total bile acid levels were measured by colorimetric assays (*n* = 6–8). (**B**) Serum taurine-conjugated primary bile acid levels were analyzed by metabolomics (*n* = 7–9). (**C**) Serum metabolomics analysis of carnitine and acylcarnitine levels (*n* = 10–12). (**D**) Serum alanine aminotransferase (ALT) levels were measured by ELISA (*n* = 11–14). RD: regular diet; HF: high-fat diet; HF.NOB: high-fat diet with 0.1% NOB. *p < 0.05, ** *p* < 0.01, *** *p* < 0.001, One-Way ANOVA; #*p* < 0.05, t-test. Bar graphs represent Mean ± SEM. For box and whisker plots, box edges correspond to 25th and 75th percentiles, lines inside of the box correspond to 50th percentiles and whiskers include extreme data points.

We next analyzed serum BA levels. Using ELISA assays, we found that the total serum BA levels increased in the HF group relative to RD; importantly, NOB treatment significantly attenuated the serum BA level at ZT6, and showed a slight trend at ZT18 (Figure 2A). We next performed serum metabolomic profiling, and the results showed concordant patterns (Figure 2B). Specifically, at ZT6, HF feeding markedly elevated BA levels relative to RD, and NOB treatment fully reversed this increase. Results at ZT18 showed similar trends but did not reach statistical significance.

It is known that a significant amount of BAs in the serum indicates leakage from the liver and is a hallmark of liver damage [48]. In addition to serum BAs, serum acylcarnitine is also indicative of liver damage, specifically as a marker of hepatic mitochondrial dysfunction [49]. Carnitine is mainly synthesized in the liver and transported to other peripheral organs through circulation as acylcarnitine. We therefore examined serum carnitine and acylcarnitine levels by metabolomics as in Figure 2B. Whereas L-carnitine levels remained constant between sample groups (Figure 2C), levels of acetylcarnitine and multiple acylcarnitines increased in the HF group (Figure 2C and Supplementary Figure S1), suggesting hepatic damage and mitochondrial dysfunction under the metabolic challenge condition. Interestingly, NOB was able to strongly reverse the increased levels of many acylcarnitine species (Figure 2C and Supplementary Figure S1). Of note, the corrective effects of NOB were greater for shorter fatty acid conjugated acylcarnitines at ZT18 and for longer fatty acid conjugated acylcarnitines at ZT6 (Figure 2C and Supplementary Figure S1).

We further examined the serum levels of hepatic alanine aminotransferase (ALT) as another established marker for liver damage. Relative to RD, the HF groups showed elevated serum ALT levels at both ZT6 and ZT18, with higher amounts found at ZT18 during the active phase when mice consumed most of their food (Figure 2D). Importantly, NOB significantly attenuated the serum ALT levels at ZT18 (Figure 2D).

### 2.3. Nobiletin Reprograms Circadian and Lipid Homeostasis Genes in the Liver

**Figure 3:**
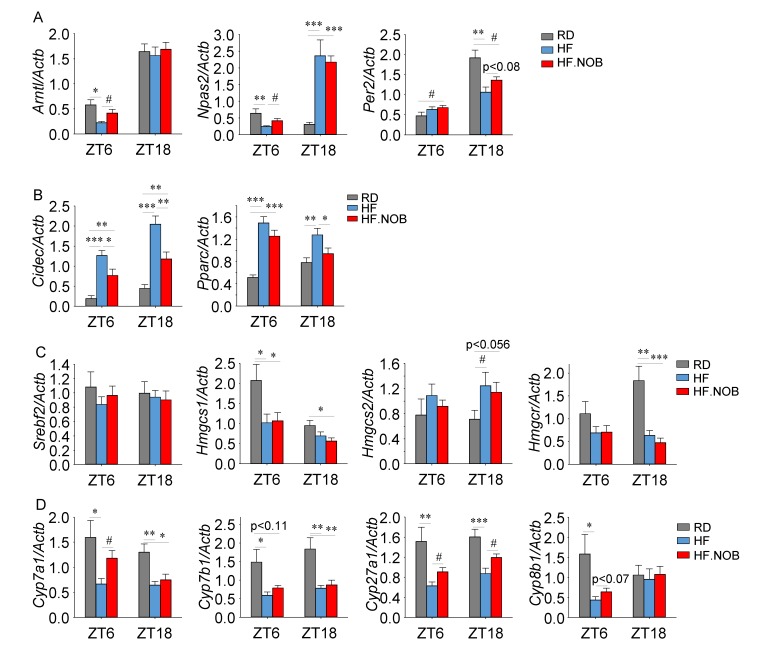
Nobiletin (NOB) reprograms the expression of circadian and lipid regulatory genes. (**A**) Core clock gene expression levels in the liver were analyzed by qPCR (*n* = 7–11). (**B**) Fatty acid and lipid metabolism related genes in the liver were analyzed by qPCR (*n* = 7–11) (**C**) Cholesterol biosynthesis related genes in the liver were analyzed by qPCR (*n* = 7–11). (**D**) Bile acid synthesis related genes in the liver were analyzed by qPCR (*n* = 7–11). RD: regular diet; HF: high-fat diet; HF.NOB: high-fat diet with 0.1% NOB. *p < 0.05, ** *p* < 0.01, *** *p* < 0.001, One-Way ANOVA; #*p* < 0.05, t-test. Bar graphs represent Mean ± SEM.

We next examined the expression of clock genes in the liver by qPCR, using liver tissues collected at ZT6 and ZT18 (Figure 3 and Supplementary Figure S2A,B). *Arntl* (*Bmal1*), *Npas2* and *Per2* genes showed significant, or a trend of, induction by NOB (Figure 3A). In our previous study [44], we identified several metabolic output genes strongly regulated by NOB [44]. Similarly, the expression of *Cidec* and *Pparg* was markedly induced by NOB treatment, while expression of *Igfbp2*, *Scd1* and *Pgc1a* showed no or minor effects (Figure 3B, Supplementary Figure S2C).

We next examined expression of *Srebf1* (de novo lipogenesis), *Elovl6* and *Ppara* (fatty acid metabolism). *Srebf1* expression did not show any change under HF or NOB treatment (Supplementary Figure S2D). *Elovl6* expression was upregulated at ZT18 by HFD and showed a trend of normalization (reduction) by NOB treatment (Supplementary Figure S2D). On the other hand, *Ppara* expression was upregulated by HFD at ZT18, but was not altered by NOB treatment (Supplementary Figure S2D).

To specifically investigate the effects of NOB on cholesterol synthesis, we determined the expression of genes in the cholesterol synthesis pathway. *Srebf2*, encoding a master regulator of sterol and fatty acid synthesis [50], did not show any changes in the HF and HF.NOB groups relative to the RD group (Figure 3C). The expression of *Hmgcs1* and *Hmgcr* genes, encoding HMG-CoA synthase and HMG-CoA reductase respectively, were reduced in the HF group but not recovered by NOB (Figure 3C). *Hmgcs2* expression was upregulated in the HF group, and NOB did not affect its expression (Figure 3C).

*Cyp7a1*, a rate-limiting enzyme in the bile acid synthesis pathway, has been shown to display robust circadian oscillatory expression regulated by RORs [38]. Several other key genes involved in bile acid synthesis, including *Cyp8b1* and *Cyp7b1*, also harbor the RORE element that is directly recognized by RORs [37,39]. Therefore, we examined mRNA expression of these potential target genes for NOB using the same liver tissues described above. Under HF feeding, expression of these BA synthesis genes were down-regulated compared with RD (Figure 3D). Importantly, NOB was able to restore the normal expression levels of several ROR target genes important for bile acid synthesis (Figure 3D), supporting the direct regulatory role of the ROR-NOB axis for BA synthesis genes.

### 2.4. Nobiletin Upregulates Fecal Bile Acids Levels

Hepatic BA synthesis from cholesterol and fecal BA excretion are important for cholesterol and bile acid. Daily hepatic production and fecal excretion of BAs are quantitatively similar, accounting for approximately 5% of total BA levels. We next examined fecal BA excretion by metabolomics, which can serve as an estimate of hepatic BA production and total BA levels. As shown in Supplementary Figure S3A–D, the overall profiles of lipids/fatty acids, while distinct between different diets, were similar between HF and HF.NOB. For example, saturated, mono- and poly-unsaturated fatty acids showed largely constant levels with or without NOB treatment (Supplementary Figure S3A–D), suggesting that NOB did not affect lipid absorption in the gut.

**Figure 4:**
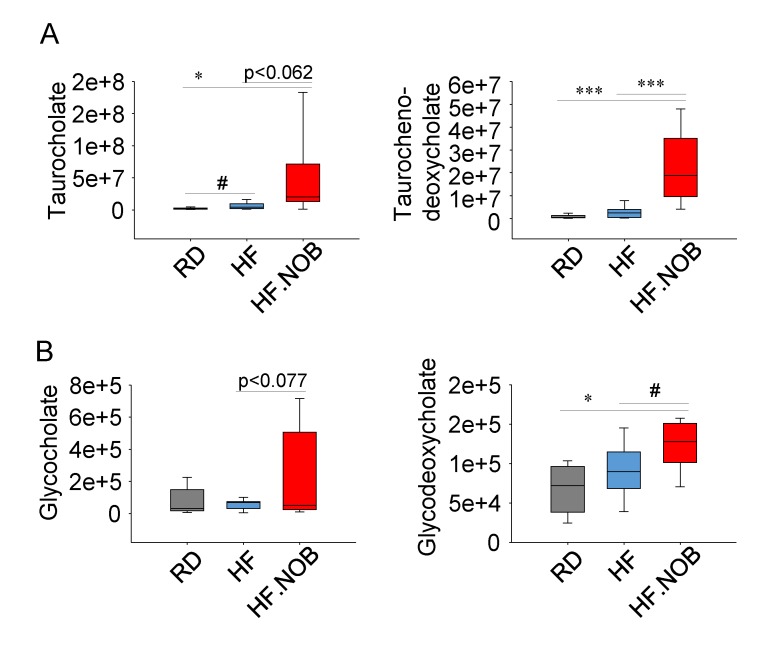
Nobiletin (NOB) enhances fecal bile acid excretion. Fecal taurine (**A**) and glycine (**B**) conjugated primary bile acid levels were measured in metabolomics (*n* = 10–12). RD: regular diet; HF: high-fat diet; HF.NOB: high-fat diet with 0.1% NOB. *p < 0.05, *** *p* < 0.001, One-Way ANOVA; #*p* < 0.05, t-test. For the box and whisker plots, box edges correspond to 25th and 75th percentiles, lines inside of the box correspond to 50th percentiles and whiskers include extreme data points.

Next, we examined the levels of taurine- or glycine-conjugated fecal primary BAs (taurocholate, taurocholate deoxycholate, glycocholate and glycodeoxycholate). Relative to RD, HF feeding elicited little changes in these BAs, with only taurocholate showing a modest increase. Interestingly however, NOB treatment significantly enhanced the levels of taurocholate deoxycholate and glycodeoxycholate, and showed a trend toward increasing the other two (Figure 4). These results further indicate the potent efficacy of NOB in BA homeostasis.

### 2.5. Nobiletin Remodels Gut Microbiota

**Figure 5:**
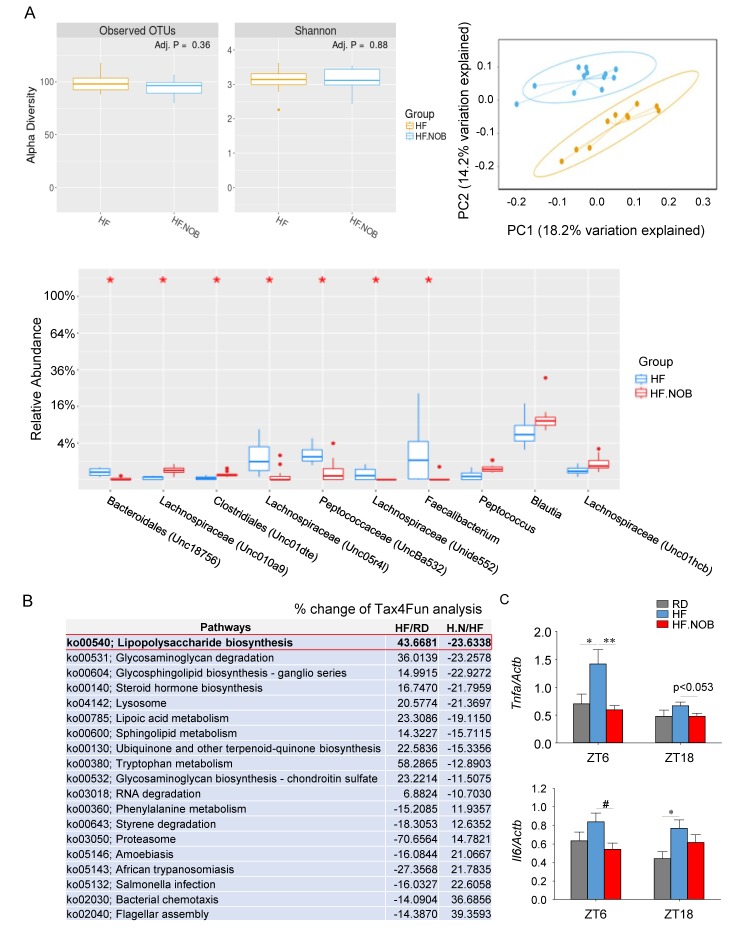
Nobiletin (NOB) remodels gut microbiota and protects the liver from inflammatory damage. (**A**) Microbial 16S rRNA sequencing showing the taxa abundance distribution at the genus level. Left top: alpha diversity box plots; right top: Principal component analysis. *P* value: 0.001; R-squared: 0.142; F-statistic: 3.3. Yellow: HF; blue: HF.NOB. Bottom: top 10 taxa abundance box plot. Panels indicate the comparison between HF and HF.NOB groups. (*n* = 10–12). (**B**) Pathway analysis using 16S sequencing data. Data indicate the comparison between HF versus RD and HF.NOB (H.N) vs HF groups. (*n* = 10–12). (**C**) Inflammatory cytokine gene expression levels in the liver were analyzed by qPCR (*n* = 7–11). RD: regular diet; HF: high-fat diet; HF.NOB: high-fat diet with 0.1% NOB. *p < 0.05, ** *p* < 0.01, One-Way ANOVA; #*p* < 0.05, t-test. Bar graphs represent the Mean ± SEM.

Gut microbiota play an important role in BA metabolism, and a number of microbial genera have been found to be involved [3,4]. We next examined whether NOB may also remodel gut microbiota by 16S rRNA sequencing (Figure 5A and Supplementary Figure S4). Analysis of taxa abundance at the genus level showed that the levels of several genera implicated in BA metabolism, e.g., *Bacteroidales* and *Peptococcus*, were significantly altered by NOB treatment (Figure 5A). These results were consistent with the regulatory role of NOB in BA homeostasis.

We further performed Tax4Fun analysis to predict functional target profiles by NOB treatment. Consistent with a significant reduction of the gram- bacteria *Bacteroidales*, we observed a reduction in lipopolysaccharide (LPS) biosynthesis in HF.NOB compared to HF (Figure 5B). LPS is known to bind to the Toll-like receptor (TLR)-4 to stimulate proinflammatory cytokine production, leading to inflammation. Consistently, expression of the two key inflammatory cytokine genes *Tnfa* and *Il6* was found to be attenuated by NOB, in support of its role in hepatic protection (Figure 5C).

## 3. Discussion

In the current study, we showed that the clock-modifying compound NOB strongly improved cholesterol and BA homeostasis in aged mice under metabolic challenge. In the serum, the LDL/VLDL levels and the LDL/HDL ratio were reduced. NOB was further shown to coordinately regulate BA homeostasis, including increased levels of fecal BA excretion, reduction of serum BA leakage, and alteration of gut microbiota involved in BA metabolism. In particular, we showed that NOB protects against liver damage and remodels hepatic gene expression involved in cholesterol/BA metabolism, including various circadian and ROR target genes. Together, this study reveals a potent role of NOB to improve cholesterol/BA homeostasis under the conditions of aging and metabolic challenge.

Our functional results were consistent with the broad physiological efficacy of NOB in metabolic homeostasis previously reported [46,51,52,53]. For example, we previously showed that NOB strongly improved glucose and lipid homeostasis in obese (HF-fed) and diabetic (*db/db*) mice, and also upregulated urea cycle activity under both high-fat and high-protein feeding conditions [44,45]. Consistent with the current work, these studies have revealed the strong beneficial effects of NOB in the liver, including attenuation of liver inflammation and remodeling of liver gene expression. For example, microarray studies unveiled the robust regulation of various lipid regulatory genes in the liver by NOB [44]. More recently, we demonstrated NOB effects in metabolic aging, and showed that NOB was able to enhance lipid homeostasis and mitochondrial respiration in the skeletal muscle of metabolically challenged aged mice [46]. Our current study further demonstrates the specific role of NOB in the cholesterol/BA metabolism, and highlights its systemic effects in the liver (both physiological markers and gene expression), serum and fecal microbiota. These studies are consistent with a broad exposure of NOB within the body and a potential application of NOB in metabolic and cardiovascular diseases. For example, in LDL receptor-deficient mice (*Ldlr(-/-)*) fed with a Western diet, NOB treatment was able to improve dyslipidemia and atherosclerosis in part via suppressing VLDL synthesis [54].

Initially identified in our circadian chemical screen, NOB was found to display a potent effect of enhancing circadian rhythms and output functions [44,55,56,57]. Importantly, using biochemical and cellular assays, we identified RORs as the direct targets of NOB [44]. The circadian mode of action was consistent with time-dependent effects of NOB in various functional assays in the current and previous studies [44,45,46,56]. Furthermore, the vast majority of regulatory genes whose expression was found herein to be modulated by NOB were also known to be clock-controlled genes [44,58]. In conjunction with significant circadian decline over age, various aspects of metabolic homeostasis also deteriorates during aging and contributes to age-related diseases [59,60,61]. For example, age-related decline in energy homeostasis can cause exaggerated lipid levels in the blood, leading to hyperlipidemia and atherosclerosis, which in turn increases the risk of myocardial infarction and stroke [62]. In a pioneering study, it was found that aging in humans was accompanied by impaired hepatic BA synthesis and a concomitantly increased excursion of cholesterol, together leading to increases in the cholesterol burden in the bile and risk of gallstones [63]. Consistent with the critical role of circadian regulation in lipid homeostasis throughout the lifespan, our study exemplifies the possible function of circadian clocks in systemic regulation of cholesterol/BA metabolism over aging, in part via ROR-regulated gene expression. Whereas small molecules, including natural compounds, often show pleiotropic effects and multiple pathway targets, future research will further dissect circadian clock-dependent and independent functions of NOB.

NOB is among a growing list of small molecules that were identified to directly target the circadian machinery [64,65,66,67,68]. A subset of these clock modifiers have been applied to disease models, and the results have shown promising effects to correct pathophysiological processes related to dysregulated clocks [69,70,71,72,73]. For example, potent agonists of REV-ERBs (SR9009 and SR9011) were recently shown to promote apoptosis of cancerous, but not normal cells, interestingly by attenuation of tumor-supporting processes including autophagy and lipogenesis [69,74]. In conjunction with these pharmacological agents, behavioral modifications (timed feeding, exercise, light exposure, sleep) and the classical chronotherapy (timed drug administration and clinical procedures) also manipulate circadian rhythms to promote health and healthy aging [75,76,77,78,79,80,81]., While temporal consideration in existing clinical trials is often observational and correlative in nature (e.g., measuring melatonin rhythms) [82], several recent examples of circadian time-dependent surgical and dietary interventions [79,83] strongly support the clinical implication of targeting the circadian system in humans. Of note, NOB is known to be generally safe and displays strong bioavailability profiles in humans, suggesting a promising application to modulate circadian and metabolic functions in translational and clinical settings [84]. Coupled with the current understanding of prevalent circadian regulation of gene expression throughout the body, including in primates [10,11], these studies underscore the circadian clock as a novel therapeutic target to safeguard health throughout our lifespan.

## 4. Materials and Methods

### 4.1. Animal Studies

Twenty two-month old mice were purchased from the National Institute on Aging (NIA). After one week of accommodation, animals were divided into three groups, namely regular diet (RD), high-fat (HF) and high-fat diet containing 0.1% Nobiletin (HF.NOB). Body weight was monitored weekly. All animal studies were approved by the UTHealth Center for Laboratory Animal Medicine and Care (CLAMC), protocol number: AWC-17-0043.

### 4.2. Serum Content Assays

Mouse serum samples were collected at the indicated circadian times (ZT6 and ZT18). Serum total bile acid levels were measured by colorimetric assay (Crystal Chem, Elk Grove Village, IL, USA). Serum total cholesterol, HDL cholesterol and LDL/VLDL cholesterol levels were measured by colorimetric assay (Abcam, Cambridge, MA, USA).

### 4.3. Real-Time qPCR

Total RNA was extracted from frozen calf muscle by TRizol (Invitrogen, Carlsbad, CA, USA). Two micrograms of extracted RNA was used for cDNA synthesis. Gene expression was analyzed by using Stratagene Mx3000p (Agilent Technologies, Santa Clara, CA, USA). Gene expression levels were normalized by *β-actin* (*Actb*) expression. Primer sequences are listed in Supplementary Table S1.

### 4.4. Metabolomic Analysis

#### 4.4.1. Sample Preparation

Collected fecal samples and serum samples were flash frozen in liquid nitrogen and stored at −80 °C until analysis. Prior to LC-MS analysis, fecal samples were placed on ice and suspended with an extraction solution containing methanol:acetonitrile:water (5:3:2, *v*:*v*:*v*) to a concentration of 15 mg/mL, while serum samples were diluted with 24 volumes of the extraction solution. Glass beads (GB10, Next Advance, Troy, NY, USA) were added to each tube and placed into a Bullet Blender (Next Advance, Troy, NY, USA) at setting 3 for 5 min at 4 °C to homogenize the tissue. Suspensions were then vortexed continuously for 30 min at 4 °C. Insoluble material was removed by centrifugation at 10,000 g for 10 min at 4 °C and supernatants were isolated for metabolomics analysis by UHPLC-MS.

#### 4.4.2. UHPLC-MS Analysis

Analyses were performed as previously published [85]. Briefly, the analytical platform employs a Vanquish UHPLC system (Thermo Fisher Scientific, San Jose, CA, USA) coupled online to a Q Exactive mass spectrometer (Thermo Fisher Scientific, San Jose, CA, USA). Samples were resolved over a Kinetex C18 column, 2.1 × 150 mm, 1.7 µm particle size (Phenomenex, Torrance, CA, USA) equipped with a guard column (SecurityGuard™ Ultracartridge—UHPLC C18 for 2.1 mm ID Columns—AJO-8782—Phenomenex, Torrance, CA, USA) using an aqueous phase (A) of water and 0.1% formic acid and a mobile phase (B) of acetonitrile and 0.1% formic acid. Either 10 μL or 20 μL of extract was injected into the system for fecal or serum samples, respectively. Samples were eluted from the column using either an isocratic elution of 5% B flowed at 250 µL/min and 25 °C or a gradient from 5% to 95% B over 1 min, followed by an isocratic hold at 95% B for 2 min, flowed at 400 µL/min and 30 °C. The Q Exactive mass spectrometer (Thermo Fisher Scientific, San Jose, CA, USA) was operated independently in positive or negative ion mode, scanning in Full MS mode (2 μscans) from 60 to 900 m/z at 70,000 resolution, with 4 kV spray voltage, 15 shealth gas, 5 auxiliary gas. Calibration was performed prior to analysis using the Pierce™ Positive and Negative Ion Calibration Solutions (Thermo Fisher Scientific). Acquired data was then converted from .raw to .mzXML file format using Mass Matrix (Cleveland, OH, USA). Samples were analyzed in randomized order with a technical mixture injected after every 15 samples to qualify instrument performance. Metabolite assignments, isotopologue distributions, and correction for expected natural abundances of deuterium, ^13^C, and ^15^N isotopes were performed using MAVEN (Princeton, NJ, USA) [86].

Graphs, heat maps and statistical analyses (either T-Test or ANOVA), metabolic pathway analysis, PLS-DA and hierarchical clustering was performed using the MetaboAnalyst 3.0 package (www.metaboanalyst.com) [87]. Hierarchical clustering analysis (HCA) was also performed through the software GENE-E (Broad Institute, Cambridge, MA, USA). XY graphs were plotted through GraphPad Prism 5.0 (GraphPad Software Inc., La Jolla, CA, USA)

### 4.5. Microbiome 16S Sequencing

Fecal samples were subjected to 16S sequencing, and data analysis was conducted by the Alkek Metagenomics Core (BCM) as previously described [88]. Tax4fun analysis was performed as previously described [89].

### 4.6. Statistical Analysis

Results were presented as mean ± sem unless otherwise stated. Data from all treatment groups were analyzed using a student’s t-test, one-way ANOVA followed by post-hoc analysis using Dunnett’s multiple comparison test or two-way ANOVA followed by post-hoc analysis using a Bonferroni test as appropriate. A value of *p* < 0.05 was considered statistically significant, and p values that fell between 0.05 and 0.1 were indicated in the figures. Sample size was determined by established standards and/or power analysis (e.g., G*Power) to detect an effect with at least 80% power, based on one-way or two-way ANOVA models at α = 0.05.

## 5. Conclusions

Emerging evidence illustrates the key regulatory role of circadian clocks in cholesterol/bile acid homeostasis. In the current study, we showed that Nobiletin (NOB), a natural compound that activates RORs and thus circadian rhythms, significantly improved serum LDL/HDL ratio, and normalized serum and fecal bile acid levels in aged mice challenged with high-fat diet feeding. In accordance, NOB was found to enhance the expression of bile acid synthesis genes in the liver, and protect overall liver health from metabolic challenge. Microbial 16S sequencing further revealed that NOB alters microbial taxa known to modulate bile acid production. Together, our work provides evidence that a pharmacological agent targeting the circadian clock strongly improves cholesterol and bile acid homeostasis in a systemic manner.

## Supplementary Materials

Supplementary materials can be found at https://www.mdpi.com/1422-0067/20/17/4281/s1.
