# Supplementary material for: Coordinate Regulation of Cholesterol and Bile Acid Metabolism by the Clock Modifier Nobiletin in Metabolically Challenged Old Mice
Source: Int J Mol Sci. 2019 Sep 1;20(17):4281. doi: 10.3390/ijms20174281 (PMC6747250; doi:10.3390/ijms20174281)
Supplement: Supplementary file 1 [file ijms-20-04281-s001.pdf]

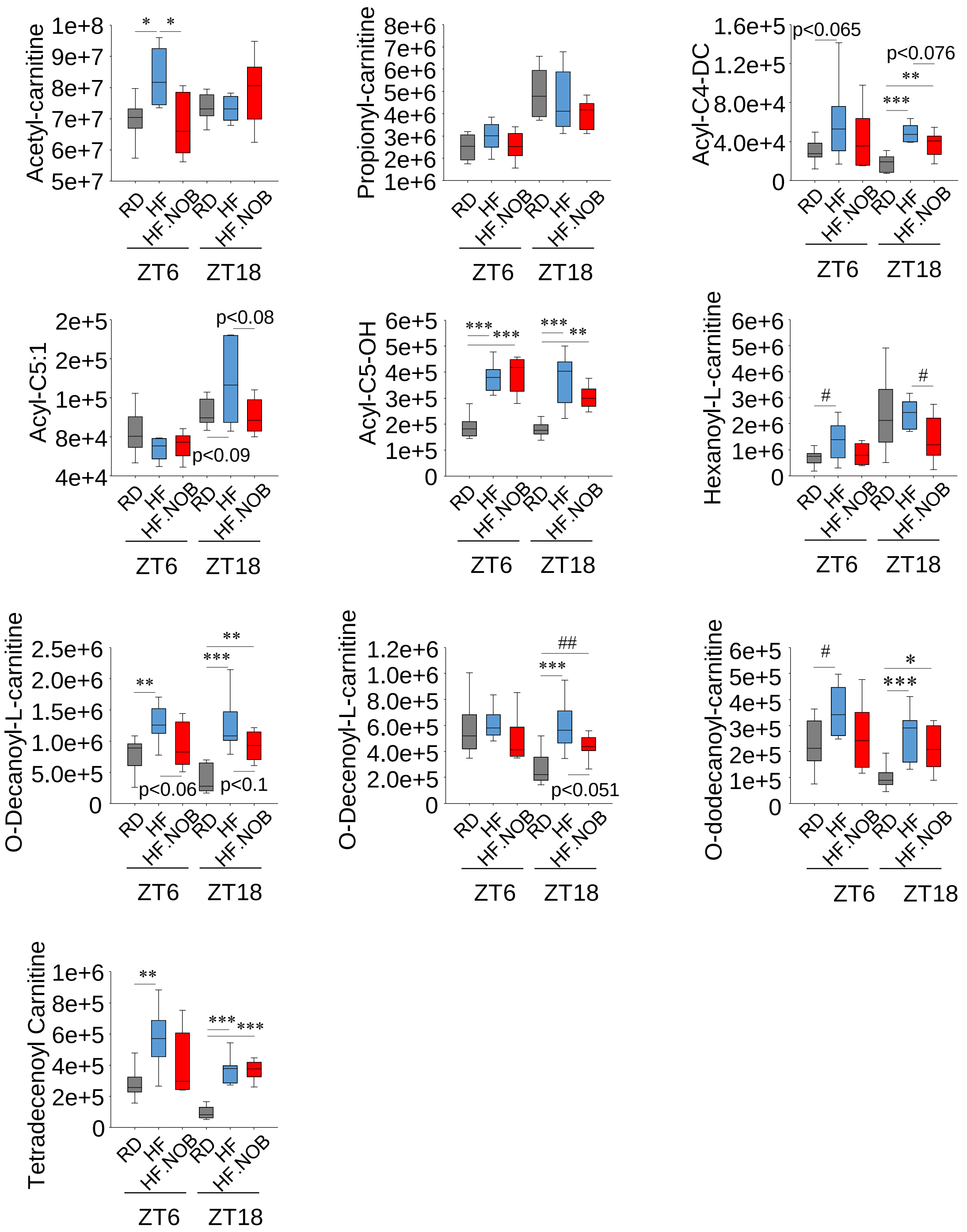


**Supplementary Figure S1. NOB reduces serum carnitine and acyl-carnitine levels.** Serum metabolomic profiles of carnitine and acyl-carnitine levels (n=7-9). RD: regular diet; HF: high-fat diet; HF.NOB: high-fat diet with 0.1% NOB. * p<0.05, ** p<0.01, *** p<0.001, One-Way ANOVA; # p<0.05, ## p<0.001, t-test. Bar graphs represent Mean ± SEM. For box-whisker plots, box edges correspond to 25th and 75th percentiles, lines inside of box correspond to 50th percentiles and whiskers include extreme data points.

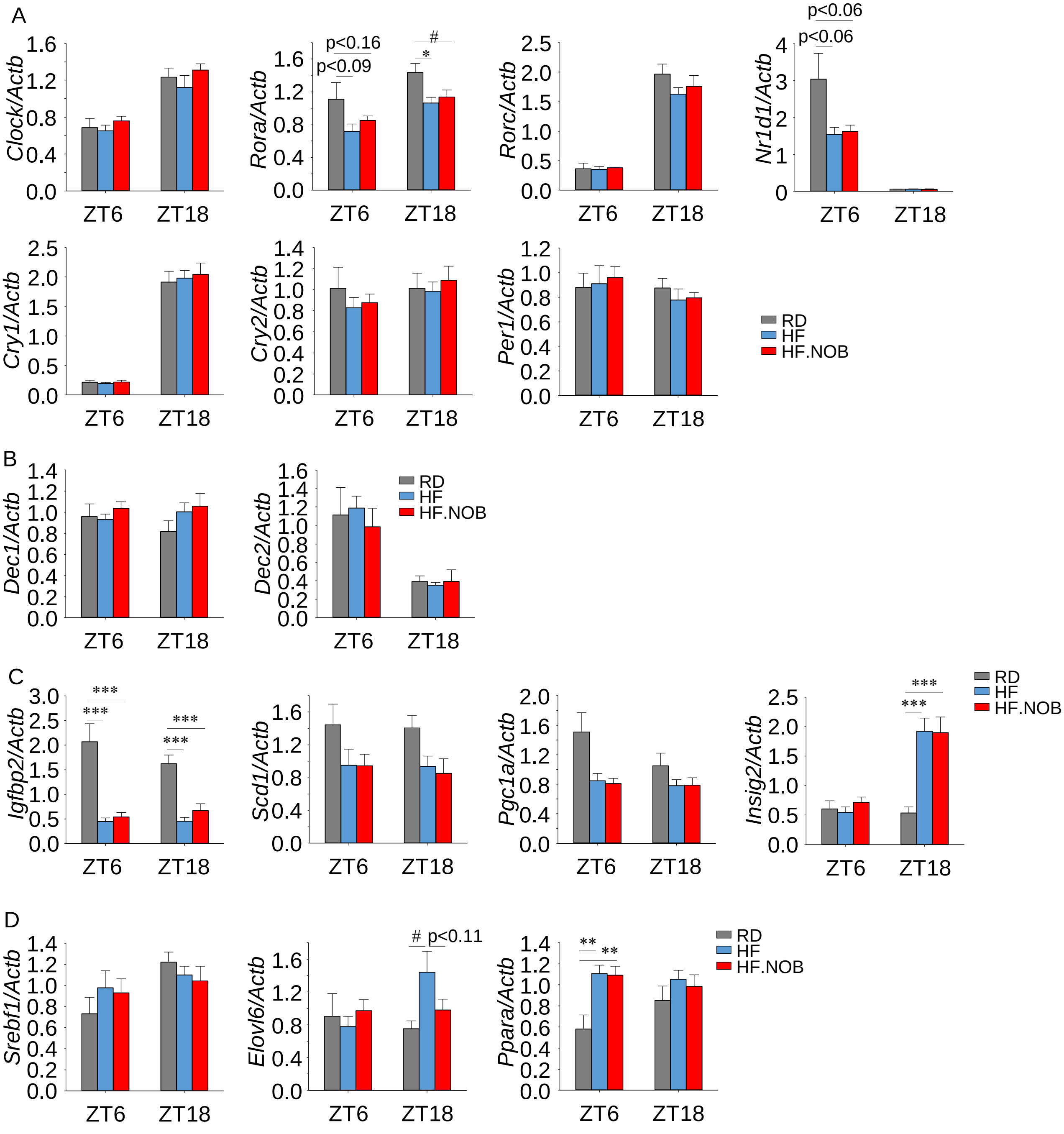


**Supplementary Figure S2. NOB alters circadian and metabolic gene expression in the liver.** (A) Core clock gene expression levels in the liver were analyzed by qPCR (n=7-11). (B) Core clock related Dec1 and Dec2 gene expression levels in the liver were analyzed by qPCR (n=7-11). (C) Fatty acid and lipid metabolism related genes in liver were analyzed by qPCR (n=7-11) (D) Clock output energy homeostasis related gene expression levels in the liver were analyzed by qPCR (n=7-11). RD: regular diet; HF: high-fat diet; HF.NOB: high-fat diet with 0.1% NOB. * $p<0.05$, ** $p<0.01$, *** $p<0.001$, One-Way ANOVA; # $p<0.05$, t-test. Bar graphs represent Mean ± SEM.

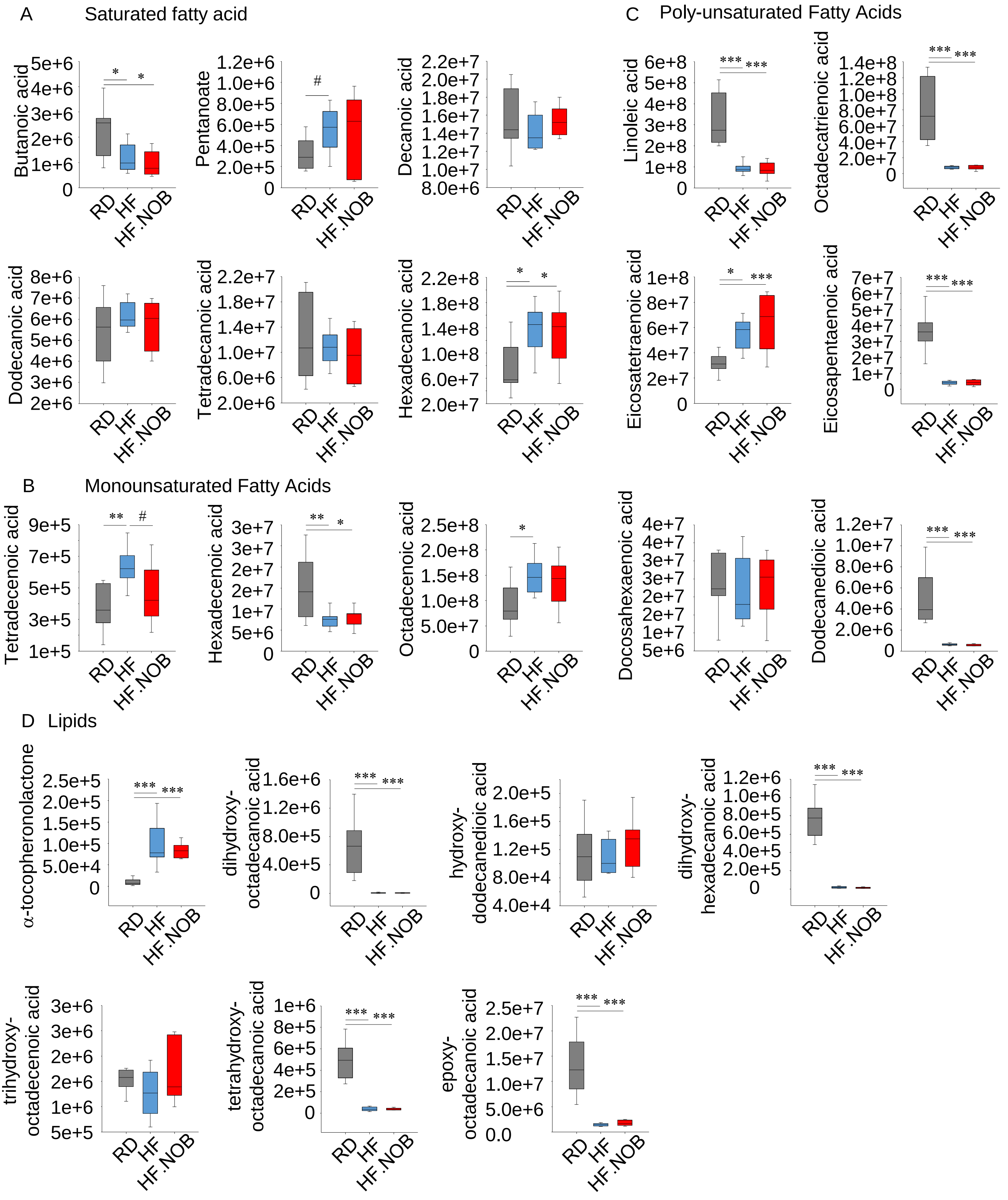

A Saturated fatty acid
C Poly-unsaturated Fatty Acids
B Monounsaturated Fatty Acids
D Lipids
Butanoic acid
Pentanoate
Decanoic acid
Dodecanoic acid
Tetradecanoic acid
Hexadecanoic acid
Linoleic acid
Octadecatrienoic acid
Eicosatetraenoic acid
Eicosapentaenoic acid
Tetradecenoic acid
Hexadecenoic acid
Octadecenoic acid
Docosahexaenoic acid
Dodecanedioic acid
α-tocopheronolactone
dihydroxy-octadecanoic acid
hydroxy-dodecanedioic acid
dihydroxy-hexadecanoic acid
trihydroxy-octadecenoic acid
tetrahydroxy-octadecanoic acid
epoxy-octadecanoic acid
RD
HF
HF.NOB

**Supplementary Figure S3. NOB does not influence fatty acid and lipid excretion.** (A) Saturated fatty acid, (B) mono-unsaturated fatty acid, (C) poly-unsaturated fatty acid and (D) other lipid profiles in fecal samples measured by metabolomics (n=10-12). RD: regular diet; HF: high-fat diet; HF.NOB: high-fat diet with 0.1% NOB. * $p<0.05$, ** $p<0.01$, *** $p<0.001$, One-Way ANOVA; # $p<0.05$, t-test. For box-whisker plots, box edges correspond to 25th and 75th percentiles, lines inside of box correspond to 50th percentiles and whiskers include extreme data points.

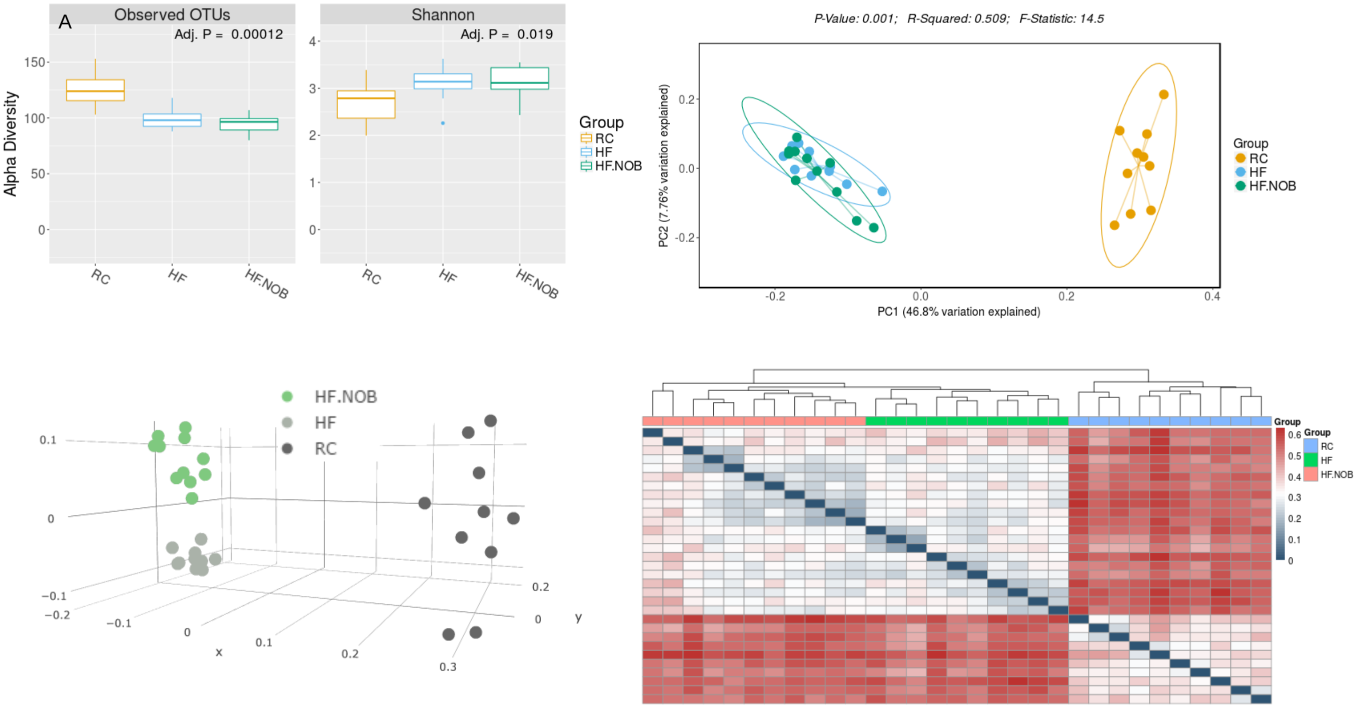


**Supplementary Figure S4. NOB remodels gut microbiota and protects liver from inflammatory damage.** Taxa abundance distribution at the genus level via 16S rRNA sequencing. Left top: box plot; right top: 2D ordination; left bottom: 3D ordination; right bottom: hierarchical plots. Panels indicate the comparison among all 3 sample groups, namely RD, HF and HF.NOB groups (n=10-12).

**Supplementary Table S1: qPCR Primer sequences for the genes listed, 5’ to 3’.**

| | Forward | Reverse |
|---|---|---|
| *Actb* | TTGTCCCCCCAACTTGATGT | CCTGGCTGCCTCAACACCT |
| *Arntl* | CCACCTCAGAGCCATTGATACA | GAGCAGGTTTAGTTCCACTTTGTCT |
| *Npas2* | CAACAGACGGCAGCATCATCT | TTCTGATCCATGACATCCGC |
| *Clock* | CCTTCAGCAGTCAGTCCATAAAC | AGACATCGCTGGCTGTGTTAA |
| *Per1* | CCCAGCTTTACCTGCAGAAG | ATGGTCGAAAGGAAGCCTCT |
| *Per2* | ATGCTCGCCATCCACAAGA | GCGGAATCGAATGGGAGAAT |
| *Cry1* | CTGGCGTGGAAGTCATCGT | CTGTCCGCCATTGAGTTCTATG |
| *Cry2* | TGTCCCTTCCTGTGTGGAAGA | GCTCCCAGCTTGGCTTGA |
| *Rora* | GCACCTGACCGAAGACGAAA | GAGCGATCCGCTGACATCA |
| *Rorc* | TCAGCGCCCTGTGTTTTTC | GAGAACCAGGGCCGTGTAG |
| *Nr1d1* | CATGGTGCTACTGTGTAAGGTGTGT | CACAGGCGTGCACTCCATAG |
| *Dec1* | GCAAGGAAACTTACAAACTGCC | CAATGCACTCGTTAATCCGGT |
| *Dec2* | ATTGCTTTACAGAATGGGGAGCG | AAAGCGCGCGAGGTATTGCAAGAC |
| *Cidec* | ATGGACTACGCCATGAAGTCT | CGGTGCTAACACGACAGGG |
| *Ppara* | AGAGCCCCATCTGTCCTCTC | ACTGGTAGTCTGCAAAACCAAA |
| *Pparc* | CGAGGACATCCAAGACAAC | TGTGACGATCTGCCTGAG |
| *Sreb1f* | CTGGCTGAGGCGGGATGA | TACGGGCCACAAGAAGTAGA |
| *Srebf2* | CACAATATCATTGAAAAGCGCTACCGGTCC | TTTTTCTGATTGGCCAGCTTCAGCACCATG |
| *Hmgcs1* | AACTGGTGCAGAAATCTCTAGC | GGTTGAATAGCTCAGAACTAGCC |
| *Hmgcs2* | ATACCACCAACGCCTGTTATGG | GTCCACATATTGGGCTGGAAA |
| *Hmgcr* | TCTTGTGGAATGCCTTGTGATT | GGGTTACGGGGTTTGGTTTAT |
| *Igfbp2* | CAGACGCTACGCTGCTATCC | CCCTCAGAGTGGTCGTCATCA |
| *Scd1* | CATCATTCTCATGGTCCTGCT | CCCATTCGTACACGTCATT |
| *Pgc1a* | TATGGAGTGACATAGAGTGTGCT | CCACTTCAATCCACCCACAAAG |
| *Insig2* | TAAATCACGCCAGTGCTAAAGT | GGTGACAACGGTTGCTAAGAAAG |
| *Elovl6* | GAAAAGCAGTTCAACGAGAACG | AGATGCCGACCACCAAAGATA |
| *Cyp7a1* | GAACCTCCTTTGGACAACGGG | GGAGTTTGTGATGAAGTGGACAT |
| *Cyp7b1* | GGAGCCACGACCCTAGATG | GCCATGCCAAGATAAGGAAGC |
| *Cyp27a1* | CCAGGCACAGGAGAGTACG | GGGCAAGTGCAGCACATAG |
| *Cyp8b1* | TGCAAAAGAACTGGTGCTCAA | CGAACCTTTAGGCCCTAGCAT |
| *Tnfa* | GCCTCTTCTCATTCCTGCTTG | CTGATGAGAGGGAGGCCATT |
| *Il6* | ACAACCACGGCCTTCCCTACTT | CACGATTTCCCAGAGAACATGTG |
